# Examination of the Knee-Jerks

**Published:** 1909-02-20

**Authors:** 


### Medicine.

### EXAMINATION OF THE KNEE-JERKS.

There are many different ways of examining the knee-jerks, some observers preferring one, some another. The patient may be either in the sitting or in the recumbent posture, and the legs may either be allowed some spontaneous position, or else be held up at the knee by the observer. Some authorities strike the ligamentum patellæ sharply with the finger tips, others with the edge of the hand, others again, with the narrow edge of some hard object, such as a thin book or a bed-letter case; whilst some will use nothing but a special hammer.

When it is merely desired to show that knee-jerks are present, they are sometimes so easily obtained that it scarcely matters what position the patient's leg is in, or how the ligamentum patellæ is percussed. In cases of doubt, on the other hand, it is most important that the best method for eliciting the jerks should be employed; and if the best method is essential in some cases it may just as well be employed in all, in order that different cases may be compared as regards the relative briskness of the jerks, and in order that the comparative readiness with which the two jerks can be elicited in the same case may also be observed. Inequality of the knee-jerks is sometimes of importance, but their inequality can only be determined when each has been tested under the most favourable circumstances for its elicitation.

If there is no objection to a patient's lying down flat upon the back on a couch or bed with the knees exposed, there is not a doubt but what this is the best posture. When the patient is lying flat in this way, the legs should be slightly flexed at the knee so that the angle behind the knee-joint is about 150°. The legs will stay in this position without being held in any way. The two knees should not be in apposition, but they should not be widely separated from one another, and their position should be perfectly symmetrical both in relation to the patient's body and in regard to the bed or couch. The heels should be resting upon the latter, the toes being just off it, and care should be taken that there is no pucker in the bedclothes or couch-cover, nor anything to impede the kick of the foot when the knee jerk is obtained. In this position it will be found that, except in cases of pathological spasticity, the calf muscles are absolutely flaccid; the quadriceps extensor femoris is just sufficiently stretched to give the necessary tonus; and a sharp transverse tap across the ligamentum patellæ will elicit the knee-jerk at once if it is present. It is important, of course, that the patient should be comfortable at the time of examination, and he should not be exposed in too chilly an atmosphere.

As to the agent employed for striking the ligamentum patellæ, there can be little doubt that the hammer is the very best of all. The force of the stroke and its position can be very accurately gauged with it, and one who has adopted the use of the percussion-hammer will not relinquish it again. Nevertheless it is quite important that, for purposes of comparison, the same observer should always use the same method; and he who has hitherto always employed the edge of a bed-letter case will probably learn more by continuing with that than by occasionally using the percussion-hammer.

If it is for any reason inconvenient or impossible to have the patient lying down, the sitting posture is the next alternative. One often sees the jerks being tested for whilst the knees are still covered by the trousers, or by pants, or by skirts and stockings. Even under these conditions it may be possible to show that the knee-jerks are present, but their proper investigation requires that the knees should be laid bare so that nothing but the skin intervenes between the striker and the ligamentum patellæ. There are three ways of dealing with the leg in which the knee-jerk is about to be tested; it may be crossed over the opposite knee as the patient sits in a chair, or it may be held up by one of the observer's hands passed under the ham-string tendons, or it may be allowed to dangle by causing the patient to sit upon the edge of a table or some other similar object which is tall enough not to allow his feet to reach the floor. Of all these three ways the commonest in general use, and at the same time the least good, is that in which the patient crosses one leg over the other. Again and again it happens that the mere fact of crossing the legs attracts the patient's attention to them to such a degree that he cannot let them "go loose," and sometimes the more he tries to do so the less he succeeds; the leg may be held so rigid that, although it is perfectly healthy, it may be impossible to obtain any knee-jerk at all. In the recumbent posture already described this difficulty very seldom arises; if the sitting posture has to be resorted to, then the best position of the leg is that in which it dangles from the edge of a table or a particularly high chair.

If there is doubt as to the presence of the knee-jerk the method of "reinforcement" should be employed. Commonly one asks the patient to grip his two hands firmly together and then to try and pull them asunder with all his might, whilst at the same time he is staring fixedly at some spot upon the ceiling.

The reason why "reinforcement" helps to elicit a knee-jerk thus is as follows: the knee-jerk, although not a true reflex, depends for its production upon a reflex tonus of the quadriceps extensor femoris muscle. This tonus is controlled by the reflex arc in the lumbar region of the spinal cord. Now the cerebrum exerts an inhibitory influence upon the spinal centres; the latter are more active when the cerebral control over them is removed. When, therefore, the major part of the voluntary or cerebral energy is being directed to the arms and eyes and deflected from the legs, there is a removal of a certain amount of inhibition from the lumbar centres of the cord, the involuntary reflex excitability of the quadriceps extensor femoris increases, and the tap upon the ligamentum patellæ will now elicit a contraction which, without this deflection of cortical activities, it was unable to do.

